# Pain and health-related quality of life in a geographically defined population of men with prostate cancer

**DOI:** 10.1054/bjoc.2001.1965

**Published:** 2001-08

**Authors:** G Sandblom, P Carlsson, P Sigsjö, E Varenhorst

**Affiliations:** Department of Urology, Faculty of Health Sciences; Centre for Assessment of Medical Technology, Linköping University, 581 85 Linköping, Sweden

**Keywords:** pain measurement, pain, quality of life, registries, prostatic neoplasms, health services accessibility

## Abstract

In order to provide baseline data on pain and health-related quality of life, to explore factors predicting pain and reduced quality of life, and to find potentially undertreated cases in men with prostate cancer, we undertook a population-based questionnaire study. The questionnaire, which included the EuroQo1 instrument, the Brief Pain Inventory form and 8 specially designed questions, was sent to all men with prostate cancer in the county of Östergötland, Sweden. Of the 1442 men included in the study, 1243 responded to the questionnaire. Altogether 42% had perceived pain during the previous week and 26% stated their quality of life to be 50% or lower on a visual analogue scale. A high rating of health care availability and short time since diagnosis were found to significantly predict lower ratings of pain (*P*< 0.05). Pain was found to be a significant predictive factor for decreased quality of life together with high age, low rating of health care availability and palliative treatment (*P*< 0.05). In conclusion, assessment and treatment of pain is essential for a good quality of life in men with prostate cancer. The monitoring of prostate cancer patients should be individualized to fit the demands of the groups with the greatest need for support. © 2001 Cancer Research Campaign http://www.bjcancer.com


*British Journal of Cancer* (2001) **85**(4), 497–503
© 2001 Cancer Research Campaign
doi: 10.1054/ bjoc.2001.1965, available online at http://www.idealibrary.com on IDEAL

http://www.bjcancer.com

### Pain and health-related quality of life in a geographically defined population of men with prostate cancer

**G Sandblom[1], P Carlsson[2], P Sigsjö[2] and E Varenhorst[1]**

[1]Department of Urology, Faculty of Health Sciences and the [2]Centre for Assessment of Medical Technology, Linköping University, 581 85 Linköping, Sweden

**Summary** In order to provide baseline data on pain and health-related quality of life, to explore factors predicting pain and reduced quality of life, and to find potentially undertreated cases in men with prostate cancer, we undertook a population-based questionnaire study. The questionnaire, which included the EuroQo1 instrument, the Brief Pain Inventory form and 8 specially designed questions, was sent to all men with prostate cancer in the county of Östergötland, Sweden. Of the 1442 men included in the study, 1243 responded to the questionnaire. Altogether 42% had perceived pain during the previous week and 26% stated their quality of life to be 50% or lower on a visual analogue scale. A high rating of health care availability and short time since diagnosis were found to significantly predict lower ratings of pain ($P < 0.05$). Pain was found to be a significant predictive factor for decreased quality of life together with high age, low rating of health care availability and palliative treatment ($P < 0.05$). In conclusion, assessment and treatment of pain is essential for a good quality of life in men with prostate cancer. The monitoring of prostate cancer patients should be individualized to fit the demands of the groups with the greatest need for support. © 2001 Cancer Research Campaign http://www.bjcancer.com

**Keywords**: pain measurement; pain; quality of life; registries; prostatic neoplasms, health services accessibility

For a long time the main goal in prostate cancer care has been to prolong the life of patients. However, with increased survival of men with prostate cancer leading to a long life after diagnosis and treatment, interest in quality of life in addition to quantity of life has increased. The therapeutic options for prostate cancer may provide control of the tumour for several years, even if the tumour is not radically treated, but the treatment as well as the tumour itself often influences general well-being in several ways, in particular in sexual and urinary functions. Metastatic spread may cause longlasting pain, although this may be avoided with palliative radiotherapy and effective analgesia (Portenoy and Lesage, 1999). However, this requires a high availability of the health care providers. If the prostate cancer care is to be effective for the whole population, the barriers to health care have to be reduced, especially for those who have the greatest difficulties in finding their way in the health care system (Mandelblatt et al, 1999).

Psychometric instruments are used for the assessment of quality of life. These typically contain questions presented as scales, each measuring one aspect of health-related quality of life. In the case of prostate cancer, mobility, urinary and sexual function, self-care, social relationships and mood are of particular interest. For patients with advanced tumours, pain and how it interferes with physical activity, sleep, and other daily functions become fundamental issues.

There have been health-related quality of life studies using psychometric tests following radical prostatectomy and radiotherapy (Pedersen et al, 1993; Krupski et al, 2000; Fowler et al, 2000; Helgasson et al, 1997), hormonal treatment (Rosendahl et al, 1999) and watchful waiting (Jönler et al, 1998) as well as for men undergoing screening for prostate cancer (Smith et al, 2000) based on selected cases under experimental conditions. Our understanding of the quality of life of men with prostate cancer has also been augmented by CaPSURE, a large national observational database of patients with prostate cancer in the USA (Lubeck et al, 1999). However, in order to be able to understand how quality of life for men in the community as a whole is affected by prostate cancer, population-based studies without selection based on treatment or hospital enrolment are required.

As regards quality of life, a knowledge of the prevalence of pain amongst all men with prostate cancer is necessary for effective management of the disease. Pain is often the most dreaded feature for patients with cancer and it is that aspect of quality of life that concerns many of them most (Wang et al, 1999). Studies have shown that doctors often underestimate the importance of pain among cancer patients and do not provide adequate analgesia (Cleeland et al, 1994; Larue et al, 1995). The presence of pain as a component of quality of life has been studied on a large scale amongst patients with advanced tumours (Cleary et al, 1995; Curran et al, 1997), but to reach a full understanding of the epidemiology of pain, not limited to advanced or terminal cases, all cancers in a population-based setting have to be studied (Greenwald et al, 1987).

The first aim of our study was to provide baseline data on the prevalence of pain in a population-based sample of men with prostate cancer. The second was to explore how health-related quality of life was affected by the disease. The third was to investigate the impact pain has on daily functions and quality of life amongst men with prostate cancer. The fourth was to evaluate factors influencing pain and quality of life amongst men with prostate cancer. The fifth was to find any potentially undertreated groups of people with pain. In order to achieve this we sent a questionnaire based on 2 validated instruments to all prevalent cases of prostate cancer in the county of Östergötland, Sweden.

*Received 22 December 2000*
*Revised 01 May 2001*
*Accepted 16 May 2001*

*Correspondence to:* G Sandblom

### MATERIAL AND METHODS

### Study base

The basic source of information was a questionnaire sent to all men with prostate cancer in the county of Östergötland. Östergötland is one of 3 counties of the South-East Health Care region (Östergötland, Jönköping and Kalmar county). The total population in Östergötland was 412 000 in 1999. It has 2 peripheral hospitals and 1 central referral hospital. All cases of prostate cancer were identified in the National Tumour Register, which was started in 1958 as a population-based cancer register and has a coverage greater than 98% (Mattsson, 1977). It contains data on all tumours diagnosed, including personal number and date of diagnosis. For cases diagnosed in 1987 or later, additional data on tumour stage, grade and treatment were extracted from the South-East Region Prostate Cancer Register, which serves as an extension of the National Tumour Register (Sandblom et al, 2000). For cases diagnosed prior to 1987, this information was achieved through a review of their case histories at each respective urology department. The central death register was searched for cases that had died before the start of the study and these were excluded. All data in the questionnaire were converted into electronic form by scanning and then checked once manually.

### Subjects

There were 7199 cases of prostate cancer diagnosed in Östergötland and registered in the National Tumour Register up until December 31 1998; of these 4474 were registered as dead in the Tumour Register. A total of 30 cases were excluded due to an incomplete personal registration number, diagnosis before 1969 or because they were born before 1900. The remaining 2695 cases were cross-linked with the National Population Register, which resulted in the further exclusion of 1145 deceased cases, 8 cases in which matching with the personal registration number was impossible to achieve and 40 cases who had moved out of the county. In a repeated matching with the population register in November 1999 an additional 60 deaths were recorded, leaving 1442 cases in the group studied.

The first letter with the questionnaire and an explaining letter was sent in September 1999. Two letters were sent 2 weeks and 4 weeks after the first letter as reminders to non-responders. A nurse was available on the telephone at each of the 3 urology departments in the county to provide general information and clarification of the questions. Of the remaining 1442 still alive at the start of the study, there were 1243 (86%) responders. The reasons for drop-out were: non-responders (145), inability to answer due to disease (34), absence of answers in the returned questionnaires (8), refusal to answer (5), not reachable at noted address (4) change of address (1) answered by wrong person (1), and obviously inaccurate answers (1). Of the 1243 responders, 78 had prostate cancer diagnosed before 1987.

The primary treatment of the 1243 responders was distributed between watchful waiting ($n$ = 582), palliative treatment including bilateral orchiectomy ($n$ = 127), GnRH-analogues ($n$ = 238), transurethral resection of the prostate (TUR-p, $n$ = 37) antiandrogens ($n$ = 15) and oestrogen ($n$ = 8), and treatment with curative intent, including radical prostatectomy ($n$ = 156), external radiation therapy ($n$ = 58) and brachytherapy ($n$ = 16). Information on treatment was missing for 6 cases. Of those initially managed with watchful waiting, 15 later received treatment with curative intent and 184 with palliative treatment. Similarly, of those who initially were treated with curative intent, 30 patients later received palliative treatment and of those initially receiving palliative treatment, 4 were later treated with curative intent. At the time of the questionnaire, 383 men were thus managed with watchful waiting; 635 patients received palliative treatment and for 219, treatment with curative intent was registered as the last treatment received.

### Questionnaire

The questionnaire was composed as a combination of the EuroQol, parts of the Brief Pain Inventory (BPI) form and 8 specially designed questions. Altogether there were 26 questions.

EuroQol is a non-disease-specific instrument for describing and evaluating health-related quality of life (EuroQol© Group, 1990). It was developed as an internationally standardized complement to other health status measures with 5 questions covering the basic domains common to generic health status and a visual analogue scale (VAS) for indication of general health state. The answers to the first 5 questions can be derived to produce an overall index of health status (EQ-5D). A validation of EuroQol in Sweden has shown a striking similarity to studies in other European centres (Brooks et al, 1991). It also has a good test–retest reliability (van Agt et al, 1994). In 1999 The EuroQol was also sent to a randomly selected group of 2700 men in the country council of Östergötland, forming a reference group to the presently studied group.

The BPI is an instrument designed to assess the severity and impact of pain on daily functions among patients with cancer pain and pain due to chronic diseases. It rates the degree to which pain interferes with mood, walking and other physical activity, work, social activity, relations with others and sleep. The BPI has been validated in several studies in which the instrument was applied to cancer patients and others who had pain (Serlin et al, 1995; Cleeland et al, 1996). It has also been shown to have respectable reliability (Cleeland and Ryan 1994). It contains 4 questions on pain severity and 7 questions on how pain interferes with daily functions.

In addition to the standardized questions from the EuroQol and BPI, 8 specially designed questions were included:

1. How would you rate your general physical condition during the previous week? (1–7)
2. How would you rate your general quality of life during the previous week? (1–7)
3. How efficient do you think your pain treatment is? (1–4)
4. Do you receive treatment in time when you have pain? (1–4)
5. Do you suffer from any side effects of the treatment? (Yes/No)
6. Which drugs do you take for pain or any other cause?
7. How difficult has it been to get access to a nurse or physician when needed the last half year? (5 grades from ‘always easy’ to ‘always difficult’ and a choice at ‘need of contact with health care’)
8. What is your marital state? (Single/Married).

### Pain management index

In order to determine whether the patient was adequately managed for his pain, a pain management index (PMI) was estimated (Fowler et al, 1995). The index was derived by subtracting the rating of most pain on the BPI questionnaire from a score

 *© 2001 Cancer Research Campaign*

corresponding to the strongest prescribed analgesic as notified by the respondent. The analgesic drug score was defined according to the World Health Organization's (WHO's) analgesic ladder: 0 for no analgesics, 1 for non-opioids, 2 for weak opioids and 3 for strong opioids. Based on the most pain as stated in the BPI questionnaire, the pain score (0–10) was categorized as 0 for no pain (rating 0), 1 for mild pain (ratings 1–3), 2 for moderate pain (ratings 4–7) and 3 for severe pain (ratings 8–10). A negative score indicates undertreatment of the pain (Cleeland et al, 1994).

### Statistics

In the analyses, localized tumours were defined as T0-2, NX/N0 and M0; all others were treated as advanced tumours. The treatment was categorized into 3 groups: watchful waiting, palliative treatment (including bilateral orchiectomy, GnRH-analogues, TUR-p antiandrogens and oestrogen) and treatment with curative intent (including radical prostatectomy, external radiation therapy and brachytherapy). In a multivariate regression analysis, factors predicting outcome from the BPI question regarding 'pain on average' in the last week (11 grades) were assessed, including patient age, marital state, time since diagnosis, presence of distant metastases at time of diagnosis, and last received and rating of health care availability, as independent variables. The treatment was categorized as palliative, curative and watchful waiting, with watchful waiting considered a reference category. The rating of health care availability was divided into 3 categories: 'no need of contact', 'easy to get contact' (always easy and usually easy to get contact) and 'difficult to get contact' (neither easy nor difficult, usually difficult and always difficult). 'No need of contact' and 'difficult to get contact' were included in the analysis and 'easy to get contact' was treated as a reference category.

Similarly, factors predicting health-related quality of life as stated on the VAS in the EuroQol questionnaire were assessed in a multivariate regression analysis, with age, marital state, time since diagnosis, tumour stage (localized/advanced), last treatment received, rating of pain on average in the last week and rating of health care availability, as independent variables. As a question about pain or discomfort is used when deriving the EQ-5D, we preferred to use the VAS scale as a measure of quality of life in order to avoid a tautology when testing how it is influenced by pain on average. The difference between the general population and the studied population in the EQ-5D score and the rating on the EuroQol VAS scale was tested with Student's *t*-test in the age intervals of 45–54 years, 55–64 years and 65–74 years. A multivariate logistic regression analysis with age, treatment, marital state, presence of distant metastases at diagnosis, time since diagnosis and rating of health care availability, was used to assess risk factors for negative PMI.

### RESULTS

The analyses presented in this report are based on the 1243 responders. The mean age of these men was 77.3 years (standard deviation (SD) 8.0 years). The mean age of those who did not answer the questionnaire was 81.6 years (SD 7.7 years). The mean interval between diagnosis and answer to the questionnaire was 5.7 years (SD 4.3 years).

### Pain

At the time of the survey, 500 men out of 1181 perceived some pain during the past week (Table 1). The most pain during the past week was stated as 'severe' by 148 men. 'Pain on average' was the pain severity question that correlated most strongly with all the pain interference questions, except for interference with sleep, which correlated more strongly with 'most pain last week'. The mean rating of pain right now, including men rating the pain as 0, was 1.8 (95% confidence interval [CI] ± 0.14) most pain past week 2.2 (± 0.17), least pain past week 1.2 (± 0.11) and pain on average 1.7(±0.13). The Cronbach alpha rating for these 4 different estimates of pain was 0.97. The ratings from the interference questions, stratified for the rating of most perceived pain last week, are shown in Table 2. The mean rating of the most pain in the past

**Table 1** BPI severity question ratings (row percentages)

| Variable | No pain (rating 0) | Mild pain (rating 1–4) | Moderate pain (rating 5–6) | Severe pain (rating 7–10) |
|---|---|---|---|---|
| Pain right now | 695 (60%) | 258 (22%) | 134 (11%) | 79 (7%) |
| Most pain last week | 680 (58%) | 184 (16%) | 159 (14%) | 143 (12%) |
| Least pain last week | 735 (63%) | 323 (28%) | 74 (6%) | 34 (3%) |
| Pain on average last week | 679 (58%) | 280 (24%) | 158 (14%) | 49 (4%) |

The ratings, ranging from 0–10, are classified as no pain (0), mild pain (1–4), moderate pain (5–6) and severe pain (7–10), (Serlin et al, 1995). Only patients answering all questions were included ($n$ = 1166).

**Table 2** Mean rating on the BP1 interference questions, ranging from 0–10 (± 95% confidence interval), stratified for rating on pain on average

| | Rating of pain on average | | | | |
|---|---|---|---|---|---|
| Factor | No pain (rating 0) | Mild pain (rating 1–4) | Moderate pain (rating (5–6) | Severe pain (rating 7–10) | Total |
| General activities | 0.08 ± 0.05 | 2.84 ± 0.26 | 5.55 ± 0.33 | 7.26 ± 0.60 | 1.80 ± 0.16 |
| Mood | 0.10 ± 0.05 | 2.58 ± 0.25 | 4.98 ± 0.38 | 5.86 ± 0.81 | 1.61 ± 0.15 |
| Walking | 0.18 ± 0.08 | 3.34 ± 0.30 | 5.81 ± 0.43 | 7.16 ± 0.80 | 2.00 ± 0.17 |
| Work | 0.09 ± 0.06 | 2.91 ± 0.32 | 5.45 ± 0.44 | 6.66 ± 0.86 | 1.71 ± 0.16 |
| Relations with other people | 0.10 ± 0.06 | 1.83 ± 0.27 | 3.44 ± 0.43 | 4.80 ± 0.98 | 1.17 ± 0.13 |
| Sleep | 0.13 ± 0.06 | 3.50 ± 0.74 | 5.21 ± 0.43 | 5.92 ± 0.88 | 1.88 ± 0.23 |
| Enjoyment of life | 0.35 ± 0.12 | 3.19 ± 0.31 | 6.04 ± 1.25 | 6.86 ± 0.88 | 2.06 ± 0.24 |

© 2001 Cancer Research Campaign week in men with distant metastases at diagnosis (M1) was 2.6 (± 0.34) and in men without known distant metastases 2.2 (± 0.09).

As the ‘pain on average’ question was found to be the BPI pain severity variable with the strongest correlation to the pain interference questions, it was chosen as the dependent variable in a multivariate regression analysis of factors predicting pain. How the different factors predict pain on average is shown in Table 3. The following 3 factors were found to be significant in predicting pain: ‘difficult to contact health care’, ‘no need to contact health care’ and time since diagnosis. The mean rating of ‘pain on average’ depending on the health care availability is shown in Table 4 (together with other outcome variables). For men who had not received any treatment at all, the mean rating on pain on average was 1.6 (95% CI ± 0.24), for those who had received treatment with curative intent 1.3 ( ± 0.29) and for those who had received palliative treatment 1.93 ( ± 0.19).

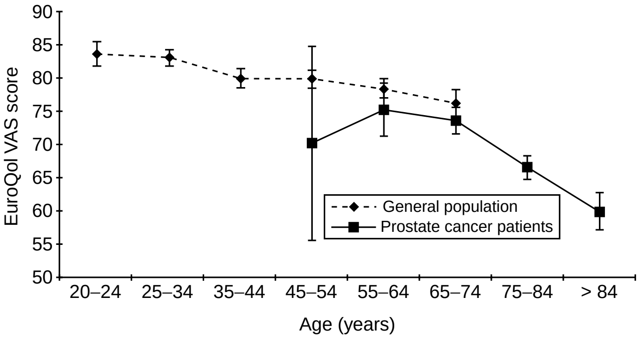

**Figure 1** Mean values on the EuroQol VAS in a sample from the total male population (Östergötland County Council questionnaire) and amongst those with prostate cancer. 95% CIs are indicated

### Health-related quality of life

The answers to the question concerning mobility varied from no problems (754), some problems (450) and confined to bed (14). Similarly concerning self-care, the answers included no problems (1059), some problems (137) and unable (28). The answers concerning usual activities were: no problems (906), some problems (216) and unable (85). Concerning pain or discomfort, the answers were: none (468), moderate (692) and extreme (60), and concerning anxiety or depression, were: none (807), moderate (393) and extreme (21). The mean ratings on the EuroQol VAS in the present study compared to the ratings from the general population in the County Council questionnaire are presented in Figure 1 and the mean EQ-5D scores in Figure 2. There was no significant difference in health-related quality of life between the general population and the men with prostate cancer, neither for EuroQol VAS or EQ-5D, in any of the age intervals from 45–74 years. Factors predicting the EuroQol VAS score in a multivariate regression analysis are shown in Table 5. As there was a high degree of co-linearity between the BPI questions, only the ‘pain on average’ variable was included in the analysis. There were 4 independent factors found to be significant: pain on average, age, difficulty in getting in contact with health care and palliative treatment. The mean scores on the EuroQol VAS scale depending on

**Table 3** Factors predicting pain on average in a multivariate regression analysis

| Factor | Standardized coefficient (beta)* | t-value | Significance |
|---|---|---|---|
| Difficult to get in contact with health care† | 0.131 | 4.307 | < 0.001 |
| No need of contact with health care† | – 0.127 | – 4.107 | < 0.001 |
| Time since diagnosis | 0.080 | 2.493 | 0.013 |
| Treatment with curative intent† | – 0.052 | – 1.426 | 0.154 |
| Palliative treatment† | – 0.025 | 0.716 | 0.474 |
| Presence of distant metastases at diagnosis† | 0.021 | 0.682 | 0.495 |
| Age | 0.019 | 0.562 | 0.574 |
| Marital status ‡ | 0.015 | 0.519 | 0.604 |

*Regression coefficient divided with the SD of each variable; †Positive = 1, Negative = 0; ‡Married = 1, Unmarried = 0.

**Table 4** Ratings of health care availability versus age, civil state and health-related quality of life as estimated by the number of men with EQ-5D scores ≤ 0.5 and the number of men who rated their quality of life on the VAS in EuroQol ≤ 50. The number of men rating severe pain (7–10) on the pain on average question (BPI) and the number of men with negative PMI are also presented

| Rating of availability | No. | Mean age (years) | Single living | Pain on average rated severe (7–10) | EQ-5D score ≤ 0.5 | EuroQol VAS ≤ 50 | Negative PMI |
|---|---|---|---|---|---|---|---|
| No need for contact with physician or nurse in past 6 months | 506 | 78 | 131 (26%) | 19 (4%) | 42 (8%) | 97 (19%) | 130 (26%) |
| Always easy/usually easy to get in contact | 573 | 77 | 128 (22%) | 25 (4%) | 55 (10%) | 147 (26%) | 178 (31%) |
| Neither easy nor difficult/usually difficult/always difficult to get in contact | 119 | 77 | 35 (29%) | 6 (5%) | 23 (19%) | 54 (45%) | 57 (48%) |
| Total | 1198 | 77 | 294 (25%) | 50 (4%) | 120 (10%) | 298 (25%) | 365 (30%) |

 © 2001 Cancer Research Campaign

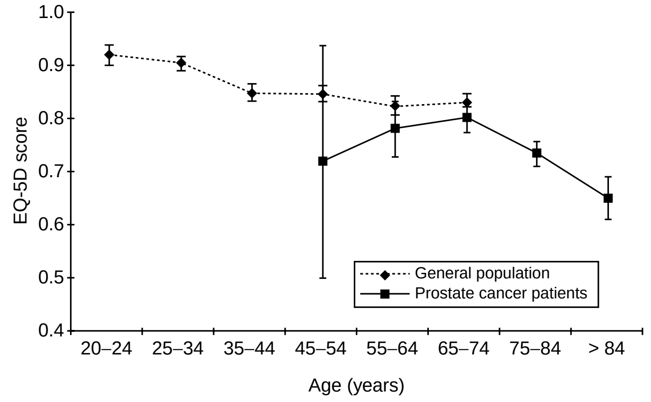

**Figure 2** Mean values on the EQ-5D score in a sample from the total male population (Östergötland County Council questionnaire) and among those with prostate cancer. 95% CIs are indicated

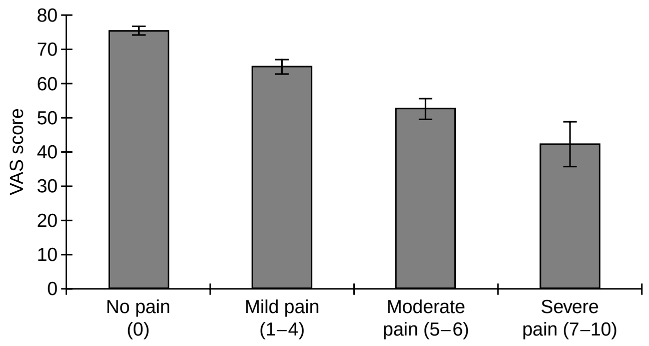

**Figure 3** Mean ratings on the EuroQoL VAS depending on the answer to the 'pain on average question'. 95% CIs are indicated the rating of health care availability are shown in Table 4. The mean score on the EuroQol VAS for patients who had not received therapy was 70 on a scale of 1–100 (95% CI ± 2). The score for those who had received palliative treatment as last therapy was 64 ± 2 and for those who had received curative treatment, 74 ± 3. The relationship between the answers to the 'pain on average' question and the rating on the EuroQOL VAS is shown in Figure 3.

### Pain treatment

Altogether 995 (81%) study participants stated that they did not receive analgesics at all, whereas 93 (8%) received non-opioids, 112 (9%) weak opioids and 35 (3%) strong opioids as the most potent form of analgesia. The corresponding figures in men with distant metastases at diagnosis (M1) were 50 (58%) for no analgesia, 5 (6%) for non-opioids, 17 (20%) for weak opioids and 14 (16%) for strong opioids. Among the 637 men who had been given palliative cancer-specific treatment, 29 (4.6%) received strong opioids. The mean EQ-5D score was 0.39 for those who received strong opioids as compared to 0.74 for the whole group.

The answers to the question concerning effect of pain treatment were distributed between very well 45 (17%), fairly well 174 (64%), rather poorly 41 (15%) and very poorly 9 (3%). Of the 272 participants who had received pain treatment, 75 (28%) stated that they suffered from some form of side effect of their medication. The answers to the question of whether pain treatment was given within reasonable time varied from: always 78 (29%), most of the time 145 (54%), seldom 33 (12%) and never 14 (5%).

Altogether 379 (30.5%) participants had a negative PMI. In 67 cases, information was not complete enough to estimate PMI. Factors predicting the risk for negative PMI in a multivariate logistic analysis are shown in Table 6. There were 3 factors found to be significant: absence of distant metastases at diagnosis (MX or MO), 'difficult in contacting health care Professionals' and 'no need to contact health care'. Of the 81 men with distant metastases at diagnosis, 14 (17.3%) had a negative PMI. Of the 364 patients who had not received therapy, 125 (34.3%) had a negative PMI. The same figure for those who had received curative treatment as last therapy was 53 out of 207 (25.6%), and for those who had received palliative treatment, 199 out of 605 (32.9%). The percentages of negative PMI depending on rating of health care availability are presented in Table 4.

**Table 6** Factors predicting negative PMI in multivariate logistic analysis

| Factor | Exp (B) | Significance |
|---|---|---|
| Difficulty in contacting health care professionals* | 0.47 | < 0.001 |
| Presence of distant metastases at diagnosis* | 2.94 | < 0.001 |
| No need of contact with health care professionals* | 1.28 | 0.082 |
| Treatment with curative intent* | 0.69 | 0.087 |
| Time since diagnosis | 1.00 | 0.48 |
| Marital status† | 1.06 | 0.73 |
| Palliative treatment* | 0.97 | 0.87 |
| Age | 1.00 | 0.91 |

*Positive = 1, Negative = 0; ‡Married = 1, Unmarried = 0.

**Table 5** Factors predicting health-related quality of life as estimated on the EuroQol VAS in a multivariate regression analysis

| Factor | Standardized coefficient (beta)* | t-value | Significance |
|---|---|---|---|
| Pain on average | – 0.436 | – 16.511 | < 0.001 |
| Age | – 0.173 | – 5.779 | < 0.001 |
| Difficulty into contacting health care professionals† | – 0.118 | – 4.370 | < 0.001 |
| Palliative treatment† | – 0.072 | – 2.367 | 0.018 |
| No need of contact with health care† | 0.042 | 1.532 | 0.126 |
| Time since diagnosis | – 0.026 | – 0.920 | 0.358 |
| Marital status‡ | 0.018 | 0.663 | 0.507 |
| Tumour stage at diagnosis§ | – 0.010 | – 0.367 | 0.714 |
| Treatment with curative intent† | – 0.007 | 0.207 | 0.836 |

*Regression coefficient divided with the standard deviation of each variable; †Positive = 1, Negative = 0; ‡Married = 1, Unmarried = 0; §Advanced = 1, Localized = 0.

© 2001 Cancer Research Campaign 

### DISCUSSION

Using the National Tumour Register with its rigorous measures to obtain complete information (Mattsson, 1977) as a base for assembling the sample, it has been possible to guarantee that the present study is essentially population based. This is further ensured by the high compliance rate of participants. The reply rate of 86% was achieved partly due to the reminder letters and nurses available on the telephone to provide assistance.

More than half of the men included in the study stated that they did not have any pain in all of the BPI pain severity answers (Table 1). The relatively small discrepancy between the 4 pain questions, designed to reflect the variations in pain intensity during the previous week, also indicate that the pain intensity remained on a stable level. However, 25% stated moderate to severe pain on some occasion during the past week, possibly representing men with refractory tumours or men with generalized tumours who had not yet received hormonal treatment. This number could probably be reduced to 10% with an increased awareness of the prevalence of pain among cancer patients and if the risk of under-reporting of pain and non-compliance is taken into consideration (Portenoy and Lesage, 1999). Although a large number of the men included in our study had generalized cancer, pain from metastases can, in most cases, be avoided if treated adequately (Portenoy and Lesage, 1999). Hormonal treatment usually has a good palliative effect on the primary tumour as well as the metastases. An association, although not significant, was also seen between age and pain. This may have been influenced by a higher prevalence of pain among older men in the general population. In a previous study carried out in the UK, 50.4% of patients randomly drawn from general practices reported chronic pain (Elliott et al, 1999). In this study, pain due to arthritis and back pain dominated. Arthritis as well as back pain of benign causes may also be widespread in the population of predominantly older men in our study.

Men with prostate cancer rated their quality of life slightly lower on the VAS than healthy men of the same age (Figures 1 and 2), although the CIs overlapped. The very wide CIs for the youngest age groups of men with prostate cancer are explained by the small sample size in these groups. In the CI in the 45–54 year age grap, there were only 7 men and in the CI in the 55–64 year age grap, only 73. The difference in rating caused by absence or presence of prostate cancer was minor in comparison with the impact of age, pain and treatment of the tumour. The quality of life for men with prostate cancer who have not received palliative treatment and who do not have pain from the cancer thus differs very little from that of men of the same age in the general population.

In a previous study it has been shown that the functions in the BPI questionnarie become impaired at a critical level of pain severity, which varies for each specific function (Cleeland, 1990). Ranking of the mean values on the pain interference questions in the present study also follows the same order (Table 2), except for interference with work, which had a relatively lower mean, and interference with walking, which had a relatively higher mean. These differences may be the result of pure coincidence, but may also reflect specific characteristics of men with prostate cancer. Very few participants are in the age group in which they might have a regular occupation, which makes interference with work become less important. Metastases from prostate cancer are most often localized to the lumbar spine and the pelvis, which may have a strong effect on the affected person's ability to walk.

The data presented in this report show that assessment and treatment of pain is essential for a good quality of life for patients with prostate cancer. This is of special importance for patients with advanced tumours, although tumour stage at diagnosis was not found to significantly influence either pain on average or quality of life as rated on the EuroQol VAS. Since the time elapsed since diagnosis was not constant, the extent of the tumour at the time of response may have differed considerably. Accordingly, in the absence of a significant association between tumour stage at diagnosis and pain rating, a significant association was found between time since diagnosis and pain rating. However, when interpreting the data, palliative treatment could be considered a surrogate measure of tumour progression. Palliative treatment is usually not given before the tumour has progressed to an advanced state or the primary tumour is causing considerable local symptoms. Consequently, palliative treatment was associated with more pain, a lower quality of life and a higher proportion of patients receiving strong opioids. The reduced quality of life associated with palliative treatment can also be the result of treatment-related symptoms, such as muscle atrophy, loss of libido, depression and osteoporosis.

The results indicate that the availability of a physician or nurse, when needed, is of very great importance for patients with prostate cancer. The rating of health care availability correlated strongly with the measures of quality of life as well as ratings of pain. A low rating for health care professional availability was also associated with a high percentage of negative PMI. However, 26% of the patients who stated that they had no need of contact with a physician or nurse still had a negative PMI. Although the patients in this group probably did not receive adequate pain treatment, they still indicated that they are content with the health care provided. This may be due to a lack of awareness among the patients that more efficient analgesics may be provided or a tendency to dissimulate. The association of a negative PMI with a high pain rating and low quality of life may partly be explained by a tendency amongst patients who feel general frustration to express discontent when answering all questions. However, of greater importance is probably the ever-increasing load on the Swedish health care system, resulting in congested outpatient departments and long waiting lists to see a specialist. Old and often disabled men with prostate cancer may often find it difficult to express their very specific needs in a health care system adapted to patients without communication problems. There was a slight tendency for older patients to find it more difficult to gain access to health care and they are more often single (Table 4). One way of making health care more available would be to delegate the follow up of men with prostate cancer to a specially trained nurse and introduce more individualized plans of follow up (Helgesen et al, 2000).

Despite the nature of prostate cancer, with the possibility of effective palliative treatment even for advanced tumours, there was a small number of men who stated that they were receiving insufficient pain treatment that was not provided in time. As expected, a low rating for health care availability was associated with a high risk for negative PMI. However, in contrast to a previous study (Cleeland et al, 1994), older age was not associated with an increased risk of a negative PMI, nor was marital status. The higher percentage of men with a negative PMI amongst those receiving palliative therapy indicates that they represent an undertreated group. Men with tumours in progress probably make up a large proportion of this group and should be given high priority when planning follow up. The paradoxical low percentage negative

 © 2001 Cancer Research Campaign

PMI in men with distant metastases at diagnosis is explained by the larger fraction of patients receiving stronger analgesia, whereas as the rating of most pain last week was essentially the same for the whole group.

### ACKNOWLEDGEMENTS

This study was possible by financial support from Astra-Zeneca in Södertälje, Sweden. Professor Kerstin Ekberg, Folkhälsovetenskapligt Centrum, Health University in Linköping, supplied data from the county council survey. We would also like to thank statistician Monika Dufmats, Magnus Husberg and Karin Sennfäldt for help with management of the files and data analysis. The high coverage rate was achieved through the support of Susanne Carnoch, Helene Marklund-Bau and Lena Thorsson, who supplied information about the study via telephone to the men included in the study. Gunnar Steineck helped with critical reading of the preliminary manuscript. The study was approved by the Data Ethics Committee of Sweden.

### REFERENCES

Brooks RG, Jendteg S, Lindgren B, Persson U and Björk S (1991) EuroQol©: health-related quality of life measurement. Results of the Swedish questionnaire exercise. *Health policy* **18**: 37–48

Cleary PD, Morrissey G and Oster G (1995) Health-related quality of life in patients with advanced prostate cancer: a multinational perspective. *Qual Life Res* **4**: 207–220

Cleeland CS (1990) Assessment of pain in cancer: measurement issues. In: Foley KM (ed.), *Advances in Pain Research and Therapy*, Vol. 16, pp 47–55. Raven Press: New York.

Cleeland CS, Gonin R, Hatfield AK, Edmonson JH, Blum RH and Stewart JA (1994) Pain and its treatment in outpatients with metastatic cancer. *N Engl J Med* **330**: 592–596

Cleeland CS and Ryan KM (1994) Pain assessment: Global use of the Brief Pain Inventory. *Ann Acad Med* **23**: 129–135

Cleeland CS, Nakamura Y, Mendoza TR, Edwards KR, Douglas J and Serlin RC (1996) Dimensions of the impact of cancer pain in a four country sample: new information from multidimensional scaling. *Pain* **67**: 267–273

Curran D, Fosså S, Aaronson N, Kiebert G, Keuppens F and Hall R (1997) Baseline quality of life of patients with advanced prostate cancer. European Organization for Research and Treatment of Cancer (EORTC), Genito-Urinary Tract Cancer Cooperative Group (GUT-CCG). *Eur Journal Cancer* **33**: 1809–1814

Elliott AM, Smith BH, Penny KI, Smith WC and Chambers WA (1999) The epidemiology of chronic pain in the community. *Lancet* **354**: 1248–1252

EuroQol© Group (1990) EuroQol© – A new facility for measurement of health related quality of life. *Health Policy* **16**: 199–208

Fowler FJ, Barry MJ, Lu Yao G, Wasson J, Roman A and Wennberg J (1995) Effect of radical prostatecomy for prostate cancer on patient quality of life: results from a Medicare survey. *Urology* **45**: 1007–1013

Greenwald HP, Bonica JJ and Bergner M (1987) The prevalence of pain in four cancers. *Cancer* **60**: 2563–2569

Helgasson AR, Adolfsson J and Steineck G (1997) Disease specific quality-of-life in men with prostate cancer: a three level epidemiological approach. *Journal of Epidemiology and Biostatistics* **2**: 213–218

Helgesen F, Andersson SO, Gustafsson O, Varenhorst E, Gobén B, Carnock S, Sehlstedt L, Carlsson P, Holmerg L and Johansson JE (2000) Follow-up of Prostate Cancer Patients by On-demand Contacts with a Specialist Nurse. *Scand J Urol Nephrol* **34**: 55–61

Jönler M, Nielsen OS and Wolf H (1998) Urinary symptoms, potency, and quality of life in patients with localized prostate cancer followed up with deferred treatment. *Urology* **52**: 1055–1062

Krupski T, Petroni GR, Bissonette EA and Theodorescu D (2000) Quality-of-life comparison of radical prostatectomy and interstitial brachytherapy in the treatment of clinically localized prostate cancer. *Urology* **55**: 736–742

Larue F, Colleau SM, Brasseur L and Cleeland CS (1995) Multicentre study of cancer pain and its treatment in France. *B Med J* **310**: 1034–1037

Lubeck DP, Litwin MS, Henning JM, Stoddard ML, Flanders SC and Carroll PR (1999) Changes in health-related quality of life in the first year after treatment for prostate cancer: results from CaPSURE. *Urology* **53**: 180–186

Mandelblatt JS, Yabroff KR and Kerner JF (1999) Equitable access to cancer services: A review of barriers to quality care. *Cancer* **86**: 2387–2390

Mattsson B (1977) *The Completeness of Registration in the Swedish Cancer Registry*. Stockholm: Stat Rep HS; Report No. 15

Pedersen KV, Carlsson P, Rahmquist M and Varenhorst E (1993) Quality of life after radical retropubic prostatectomy for carcinoma of the prostate. *Eur Urol* **74**: 2520–2532

Portenoy RK and Lesage P (1999) Management of pain. *Lancet* **353**: 1695–1700

Rosendahl I, Kiebert GM, Curran D, Cole BF, Weeks JC, Denis LJ and Hall RR (1999) Quality-adjusted survival (QTWiST) analysis of EORTC trial 30853: comparing goserelin acetate and flutamide with bilateral orchiectomy in patients with metastatic prostate cancer. European Organization for Research and Treatment of Cancer. *Prostate* **38**: 100–109

Sandblom G, Dufmats M, Nordenskjöld K and Varenhorst E (2000) Prostate cancer trends in three counties in Sweden 1987–1996. Results from a population-based register. *Cancer* **88**: 1445–1453

Serlin RC, Mendoza TR, Nakamura Y, Edwards KR and Cleeland CS (1995) When is cancer pain mild, moderate or severe? Grading pain severity by its interference with function. *Pain* **61**: 277–284

Smith DS, Carvalhal GF, Schneider K, Krygiel J, Yan Y and Catalona WJ (2000) Quality-of-life outcomes for men with prostate carcinoma detected by screening. *Cancer* **88**: 1454–1463

Van Agt HME, Essink-Bot ML, Krabbe PFM and Bonsel GJ (1994) Test-retest reliability of health state valuations collected with the EuroQol questionnaire. *Soc Sci Med* **39**: 1537–1544

Wang XS, Cleeland CS, Mendoza TR, Engstrom MC, Liu S, Xu G, Hao X, Wang Y and Ren XS (1999) The effects of pain severity on health-related quality of life: a study of Chinese cancer patients. *Cancer* **86**: 1848–1855

© 2001 Cancer Research Campaign
